# Prevalence and determinants of pulmonary hypertension in a group of Cameroonian patients without chronic lung disease: a cross-sectional echocardiographic study

**DOI:** 10.1186/s13104-017-2903-3

**Published:** 2017-11-07

**Authors:** Ahmadou M. Jingi, Jean Jacques Noubiap, Aurel T. Tankeu, Liliane Mfeukeu-Kuate, Clovis Nkoke, Philippe Kamdem, Alain Patrick Menanga, Samuel Kingue

**Affiliations:** 1 Department of Internal Medicine and Specialties, Faculty of Medicine and Biomedical Sciences, University of Yaoundé 1, Yaoundé, Cameroon; 2 Department of Medicine, University of Cape Town and Groote Schuur Hospital, Cape Town, 7295 South Africa; 3 Cardiology Unit, Department of Medicine, Yaoundé General Hospital, Yaoundé, Cameroon; 4 Centre Médical de la Trinité, Bafoussam, Cameroon

**Keywords:** Pulmonary hypertension, Echocardiography, Prevalence, Determinants, Cameroon, Sub-Saharan Africa

## Abstract

**Objective:**

To study prevalence and determinants of pulmonary hypertension (PH) in a group of Cameroonian patients without chronic lung disease. We conducted a cross-sectional study conducted between April and December 2011 in a private cardiology clinic in Bafoussam, Cameroon. We included consenting participants aged ≥ 18, who underwent a Doppler echocardiography. Patients with chronic lung disease were excluded.

**Results:**

A total of 178 participants were enrolled, of whom 44.4% were males with a mean age of 63.1 ± 17.3 years. The prevalence of PH was 25.3%. Among patients with PH 44.4% had severe disease, (11.2% of study population). Age ≥ 55 years, systolic blood pressure ≥ 140 mmHg, low left ventricular ejection fraction (< 55%), left atrial enlargement, left ventricular hypertrophy and presence of left heart disease (left ventricular hypertrophy with systolic dysfunction and left atrial enlargement) were predictors of echocardiography PH. Obesity was negatively associated with pulmonary hypertension. Pulmonary hypertension is found in a quarter of the participants. Age, systolic hypertension, and any left heart disease were strongly associated to pulmonary hypertension.

**Electronic supplementary material:**

The online version of this article (10.1186/s13104-017-2903-3) contains supplementary material, which is available to authorized users.

## Introduction

Pulmonary hypertension (PH) is a condition characterized by an increase in pulmonary vascular resistance, or pulmonary venous pressure, pulmonary blood flow or both [1]. It is defined as an increase in mean pulmonary arterial pressure (PAPm) ≥ 25 mmHg at rest as assessed by right heart catheterization. However, two-dimensional Doppler echocardiography is the most useful imaging modality in patients with suspected pulmonary hypertension [2]. This condition is associated with high morbidity and mortality in sub-Saharan Africa (SSA) [1, 3]. Its appears to be have higher burden in Africans, even in those in the diaspora suggesting a strong genetic predisposition in addition to the high prevalence of risk factors [4]. It is usually associated with left heart failure [3, 5–7] and chronic kidney disease [8–10] and an impaired quality of life [11]. Despite this high burden in SSA, data on PH are scarce in this region, accounting for less than one percent of the publications on the topic [12]. For instance, the still ongoing Pan African Pulmonary Hypertension Cohort (PAPUCO) study is the first ever in SSA designed to study the spectrum of etiologies and outcomes of PH [12]. In order to throw more light on the epidemiology of PH in SSA, we investigated the prevalence and determinants of PH in patients without primary lung disease in Cameroon.

## Main text

### Methods

This study is reported in accordance with the Strengthening the Reporting of Observational studies in Epidemiology (STROBE) guidelines [13].

#### Study design, setting and participants

We conducted a cross-sectional study from April to December 2011 in a private cardiologic clinic, “Centre Médical de la Trinité”, located in a semi-urban setting in Bafoussam, Cameroon. Considering that data on the topic are very scarce in our setting and context, despite the still ongoing PAPUCO, there is a need of epidemiological data for a better understanding of this condition in sub Saharan Africa. Given that, these data collected in 2011 (6 years ago) remain of great importance in order to provide a picture of the situation of this pathology in a sub Saharan African setting such as Cameroon. We included all patients aged 18 years or more seen at the clinic, consenting to participate and who underwent a Doppler echocardiography. Though pulmonary artery catheterization is the gold standard for the diagnosis of pulmonary hypertension, a doppler echocardiography was used to this purpose in the present study since a strong correlation between the two tests has been reported. In addition echocardiography is non-invasive, therefore, it is more suitable for epidemiological investigations such as this latter.

We excluded patients presenting chronic lung disease such as chronic obstructive pulmonary disease or asthma, and all factors that could influence the estimate of left ventricular mass such as septal dyskinesia on echocardiography, asymmetric septal hypertrophy (defined as septum/posterior wall ratio > 1.3) as well as large pericardial effusion or chest deformity.

#### Procedure

Consenting participants rested for 10 min during which a brief history was obtained. Then, their blood pressure was measured on both arms using a mercury sphygmomanometer with a standard cuff. A second blood pressure measurement was made in the arm with the highest reading after a further 5 min of rest, and the average of the measurements from this arm was considered. Secondly, anthropometric measurements were carried out. Weight was measured in light clothed subjects to the nearest 0.5 kg with a clinical Seca^®^ scale balance; height was measured in the upright position to the nearest 0.5 cm. Finally, trans-thoracic echocardiography was performed with the patient in the left lateral decubitus position by an experienced cardiologist (PK) with a commercially available echocardiography equipment (HP Sonos 2000 Color Doppler ver. A.2, HP Color) and using a 4–7 MHz transducer.

#### Measurements

Systolic blood pressure measurements ≥ 140 mmHg and/or diastolic blood pressure measurements ≥ 90 mmHg were considered diagnostic of hypertension [14]. Participants on anti-hypertensive drugs were also considered as having hypertension. The body mass index (BMI) in kg/m^2^ was calculated as $weight \left( {kg} \right)/\left[ {height \left( m \right) \times height\left( m \right)} \right]$. Participants with a BMI ≥ 30 kg/m^2^ were classified as obese.

Left ventricular measurements were done on long parasternal long axis 2-D guided M-mode using the ASE recommendations [15]. The LVM was calculated using the formula:

$$\begin{aligned} LVM & = 0.8 \, \left( {1.04\left[ {\left( {IVSd + LVPWd + LVEDd} \right)^{3} {-} LVEDd^{3} } \right]} \right) \\ & \quad + 0.6\;{\text{g}} \end{aligned}$$

LVM: left ventricular mass, IVSd: septal thickness in end diastole, LVPWd: posterior wall thickness in end diastole, LVEDd: left ventricular chamber size in end diastole.

LVH was defined based on ASE recommendations as Indexed LVM (LVM/m^2^ of BSA or LVM/m^2.7^ of height) > 115 g/m^2^ (BSA) or > 48 g/m^2.7^ (height in case of obesity) in men and Indexed LVM > 95 g/m^2^ or > 44 g/m^2.7^ in women [15].

Pulmonary arterial systolic pressure (PASP) was calculated using the gradient across the regurgitant tricuspid valve plus the right atria pressure. This was estimated from the peak velocity of the trans-tricuspid jet recorded by CW Doppler ultrasound. Pulmonary arterial systolic pressure is equal to right ventricular systolic pressure in the absence of pulmonary stenosis. This was estimated by calculating the right ventricular to right atrial pressure gradient during systole, approximated by the modified Bernoulli equation as 4v^2^, where v is the peak velocity of the tricuspid regurgitation jet in m/s. Right atrial pressure, estimated on the basis of echocardiographic characteristics of the inferior vena cava and assigned a standardized value. A resting PASP ≥ 35 mmHg was considered diagnostic of PH. The average of at least two measurements was used for the calculations [16].

#### Statistical methods

A sample size of 138 participants was required, assuming the prevalence of 10% of pulmonary hypertension among patients in Cameroon [12], a 5% margin of error and a 95% confidence level. Data were analyzed using the Statistical Package for Social Sciences (SPSS) version 16.0 for Windows (SPSS, Chicago, Illinois, USA). We described continuous variables using means with standard deviations (SD), and categorical variables using their frequencies and percentages with 95% confidence intervals (CI). We calculated the odds of having PH and adjusted for systemic arterial hypertension, left ventricular (LV) anomaly (hypertrophy, systolic dysfunction, diastolic dysfunction with raised filling pressure), left atrial enlargement (LAE), age, sex, and BMI. A *p* value less than 0.05 was considered statistically significant.

### Results

**Fig. 1:**
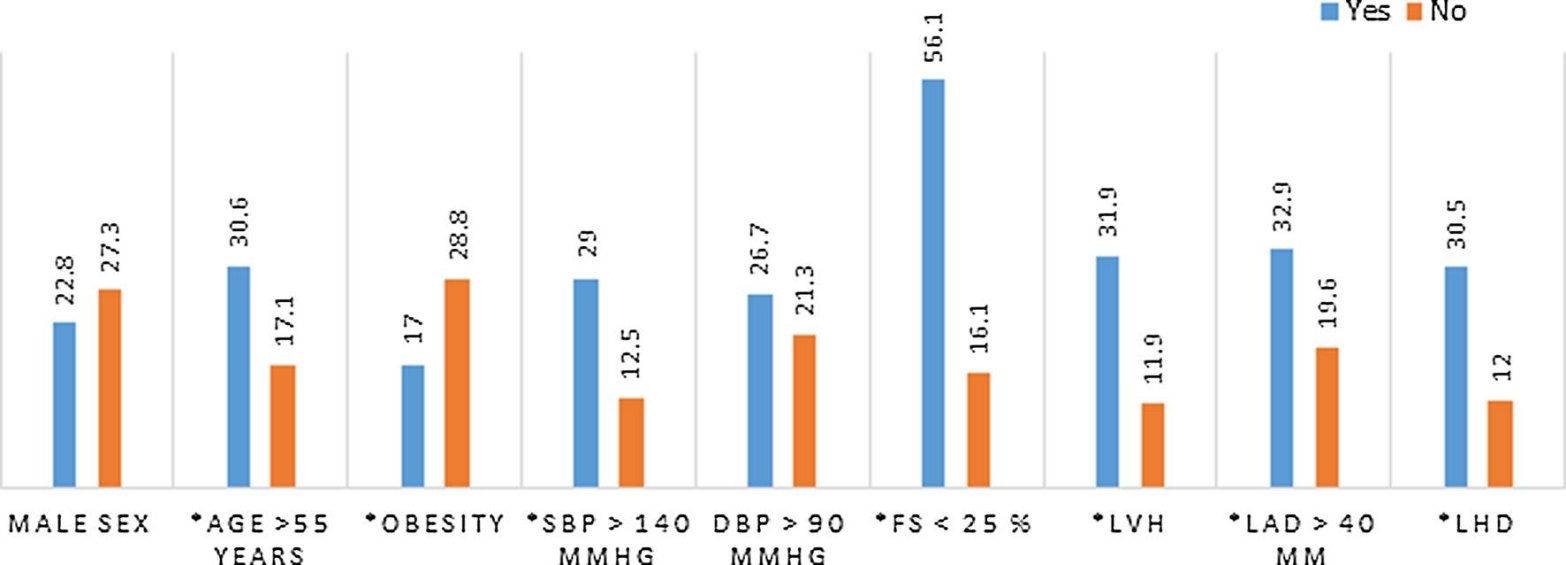
Prevalence of pulmonary hypertension according to age, gender, blood pressure and cardiac status. (*) significant

One hundred and seventy-eight (178) participants were enrolled, of whom 79 (44.4%) were males. Their mean age was 62.8 ± 15.9 years, and the age group greater than 70 years was the most frequent (37.1%). The clinical and echocardiography characteristics of the study population are summarized in Tables 1 and 2. Males had a higher atrial size and higher ventricular mass (Table 2). The prevalence of PH was 25.3% of which 44.4% had severe disease (RVSP > 55 mmHg), representing 11.2% of the study population. The prevalence of PH by age, sex, blood pressure, and left heart disease is shown in Fig. 1 and Additional file 1: Table S1. PH is more frequent in those with low ejection fraction than any other factor. Of those with PH (n = 45), 68.9% had left atrial enlargement, 84.4% had left ventricular hypertrophy including mild forms, and 71.1% had low LV ejection fraction. Table 2 summarizes the determinants of PH in the study population. Age ≥ 55 years (adjusted odd ratio (aOR) 3.17, 95% CI 1.05–9.6; *p* = 0.014), SBP ≥ 140 mmHg (aOR 4.23, 95% CI 1.31–13.6; *p* = 0.032), low LV ejection fraction (< 55%) (aOR 7.1, 95% CI 3.2–15.8; *p* < 0.001), left atrial enlargement (aOR 3.72, 95% CI 1.7–8.17; *p* = 0.002), left ventricular hypertrophy (aOR 2.68, 95% CI 1.1–6.53; *p* = 0.011), and the presence of any left heart disease (LVH with systolic dysfunction and LAE) (aOR 10.7, 95% CI 4.6–24.6; *p* < 0.001) were predictors of echocardiography PH. Obesity (BMI ≥ 30 kg/m^2^) was negatively associated with (aOR 0.38, 95% CI 0.13–1.09; *p* = 0.032) echocardiography PH. The differences in the prevalence and determinants of pulmonary hypertension in young and old female and male patients and the determinants of pulmonary hypertension in the whole population are summarized in Additional file 2: Table S2 and Additional file 3: Table S3 respectively.

**Table 1:** Clinical and echocardiographic characteristics of the study population (percentage with 95% confidence interval)

Characteristics	Percentage (95% confidence interval)		
	Overall (N = 178)	Males (n = 79)	Females (n = 99)
Age ≥ 55 years	74.7 (67.7–80.9)	74.7 (63.6–83.8)	74.7 (65–82.9)
Obesity (body mass index ≥ 30 kg/m^2^)	30.3 (23.7–37.7)	21.5 (13.1–32.2)	36.4 (26.9–46.6)
Systolic blood pressure ≥ 140 mmHg	77.5 (70.7–84.3)	82.3 (72.1–90)	73.7 (63.9–82.1)
Diastolic blood pressure ≥ 90 mmHg	73.6 (66.5–79.9)	81 (70.6–89)	67.7 (57.5–76.7)
Pulmonary hypertension (RVSP > 35 mm)	25.3 (19.1–32.3)	22.8 (14.1–33.6)	27.3 (18.8–37.1)
Left atrium diameter > 40 mm	42.7 (35.3–50.3)	51.9 (40.4–63.3)	35.4 (26–45.6)
Ejection fraction < 55%	35.4 (28.4–42.9)	41.8 (30.8–53.4)	30.3 (21.5–40.4)
Echo LVH indexed for height (g/m^2.7^)	72.5 (65.3–78.9)	67.1 (55.6–77.3)	76.8 (67.2–84.7)
Echo LVH indexed for body surface area (g/m^2^)	66.9 (59.4–73.4)	64.6 (53–75)	68.7 (58.6–77.6)

*RVSP* right ventricular systolic pressure, *LVH* left ventricular hypertrophy

**Table 2:** Clinical and echocardiographic characteristics of the study population (mean ± standard deviation)

Characteristics	Mean ± standard deviation			*p* value
	Overall (N = 178)	Males (n = 79)	Females (n = 99)	
Age (years)	62.8 ± 15.9	61.8 ± 15.9	63.6 ± 16	0.449
Body mass index (kg/m^2^)	27.5 ± 5.8	26.6 ± 5.4	28.2 ± 6	0.063
Systolic blood pressure (mmHg)	161.1 ± 33	165.1 ± 33.2	158 ± 32.6	0.158
Diastolic blood pressure (mmHg)	97.4 ± 20	98.6 ± 20.3	96.5 ± 20.1	0.491
Pulmonary hypertension (RVSP > 35 mm)	42.8 ± 17.9	39.9 ± 16	44.9 ± 19	0.226
Left atrium diameter (mm)	39.5 ± 8.5	41.5 ± 8.4	38 ± 8.4	*0.007*
LV ejection fraction (%)	56.2 ± 15.8	54.4 ± 16.6	57.7 ± 15	0.167
Echo LVH indexed for height (g/m^2.7^)	61.3 ± 21.7	62.4 ± 21.7	60.4 ± 21.9	0.552
Echo LVH indexed for body surface area (g/m^2^)	123.3 ± 46	134.4 ± 47.1	114.5 ± 43.2	*0.004*

*RVSP* right ventricular systolic pressure, *LVH* left ventricular hypertrophy

### Discussion

This study shown that pulmonary hypertension (PH) is found in a quarter of the participants without chronic lung disease in this group of sub-Saharan Africans. Age > 55 years, systolic hypertension, and any left heart disease were predictors of PH.

The evidence from the few reports on PH in SSA showed that PH is more prevalent and carries a poor disease course [3]. We have shown that systolic blood pressure was highly predictive of pulmonary pressure despite the difference in pressure regime in both systems. This suggests that the pathophysiological mechanisms in the genesis of systemic systolic hypertension equally operates in the pulmonary vasculature in the absence of any primary lung disease. Systemic hypertension is highly prevalent in SSA than in other regions [17]. Based on our findings, the higher rates of PH in SSA is an expected finding. Systemic diastolic and related pressures (mean blood pressure pulse pressure) were not shown to be determinants of PH (systolic pulmonary pressure) suggesting quite distinct pathophysiological mechanisms. Age, left atrial enlargement and left ventricular systolic/diastolic dysfunctions have consistently been shown to be strong predictors of PH in line with our findings [4, 8]. Left ventricular hypertrophy was shown to be a marginal determinant of PH. Obesity appeared protective while sex was not a determinant of PH. This contrasted with reports of Choudhary et al. [4] in a comparable population but in distinct geographical settings as well as those of Guglin et al. [18] in a young adult population. This could be due to the fact that obesity is an important feature of the obesity-hypoventilation syndrome [19]. Clinical implications are the need to increase sensitization for both the health care personnel and the general population on the necessity of a systematic screening for pulmonary hypertension in individuals aged over 55 years old especially those already presenting high blood pressure or any left heart disease.

In conclusion, PH was seen in a quarter of the participants without chronic lung disease in this group of sub-Saharan Africans. Age ≥ 55 years, systolic hypertension, and any left heart disease were strongly associated with PH. This underscores the need for screening for PH in hypertensive patients in SSA age ≥ 55 years.

### Limitations

An important limitation of this study was the used of clinical information to exclude chronic lung disease. Also, the data presented in this article were collected in 2011 (6 years ago) what makes them a bit outdated. However, despite the still ongoing PAPUCO, data on the topic are scarce especially in our setting and context and there is a need of epidemiological data for a better understanding of this condition in sub Saharan Africa. Given that, our report remain of high importance. Another limitation is the used of echocardiography which is not the gold standard for the diagnosis of pulmonary hypertension.

## Additional files

**Additional file 1: Table S1.** Prevalence and risk factors of pulmonary hypertension in males and females.

**Additional file 2: Table S2.** Prevalence and risk factors (unadjusted and adjusted) of pulmonary hypertension in the general population.

**Additional file 3: Table S3.** Determinants of pulmonary hypertension without chronic lung disease.

## Abbreviations

aOR: adjusted odd ratio
BMI: body mass index
BP: blood pressure
BSA: body surface area
IVSd: septal thickness in end diastole
LAE: left atrial enlargement
LVEDd: left ventricular chamber size in end diastole
LVH: left ventricular hypertrophy
LVM: left ventricular mass
LVPWd: posterior wall thickness in end diastole
PASP: pulmonary arterial systolic pressure
PH: pulmonary hypertension
RVSP: right ventricular systolic pressure
SSA: sub-Saharan Africa
