# Supplementary material for: Prevalence and determinants of pulmonary hypertension in a group of Cameroonian patients without chronic lung disease: a cross-sectional echocardiographic study
Source: BMC Res Notes. 2017 Nov 7;10:571. doi: 10.1186/s13104-017-2903-3 (PMC5678771; doi:10.1186/s13104-017-2903-3)
Supplement: Supplementary file 1 — Additional file 1: Table S1. Prevalence and risk factors of pulmonary hypertension in males and females. [file 13104_2017_2903_MOESM1_ESM.rtf]

Additional file 1: Table S1: Prevalence and risk factors of pulmonary hypertension in males and females
Variable	Males		Females	
	Prevalence of PH, (%, 95% CI)	OR (95% CI)	p value		Prevalence of PH, (%, 95% CI)	OR (95% CI)	p value	
Age								
≥ 55 years	27.1 (16.4 – 40.3)	3.4 (0.7 – 16.1)	0.098		32.4 (22 – 44.3)	3.5 (0.95 – 12.9)	0.038	
<55 years	10 (1.2 – 31.7)	1			12 (2.5 – 31.2)	1		
BMI 								
≥ 30 kg/m2	11.8 (1.5 – 36.4)	0.38 (0.07 – 1.86)	0.187		19.4 (8.2 – 36)	0.52 (0.19 – 1.38)	0.186	
<30 kg/m2	25.8 (15.5 – 38.5)	1			31.7 (20.6 – 44.7)	1		
Systolic BP								
≥ 140 mmHg	23.1 (13.5 – 35.2)
	1.1 (0.27 – 4.5)	0.602		34.2 (23.5 – 46.3)	6.3 (1.37 – 28.6)	0.004	
<140 mmHg	21.4 (4.7 – 50.8)	1			7.7 (0.9 – 25.1)	1		
Diastolic BP								
≥ 90 mmHg	23.4 (13.8 – 35.7)	1.22 (0.31 – 4.9)	0.538		29.9 (19.3 – 42.3)	1.52 (0.57 – 4.1)	0.405	
<90mmHg	20 (4.3 – 48.1)	1			21.9 (9.3 – 40)	1		
Pulse pressure								
≥ 65 mmHg	16.2 (6.2 – 32)	0.48 (0.16 – 1.46)	0.191		31.7 (18.1 – 48.1)	1.46 (0.6 – 3.6)	0.405	
<65 mmHg	28.6 (15.7 – 44.6)	1			24.1 (13.9 – 37.2)	1		
Mean BP 								
≥ 150 mmHg	11.5 (2.4 – 30.2)	0.33 (0.1 – 1.27)	0.08		31.3 (16.1 – 50)	1.34 (0.52 – 3.4)	0.539	
<150 mmHg	28.3 (16.8 – 42.3)	1			25.4 (15.5 – 37.5)	1		
Left Atrial Enlargement								
Yes	36.6 (22.1 – 53.1)	6.7 (1.8 – 25.7)	0.002		45.7 (28.8 – 63.4)	4.1 (1.6 – 10.3)	0.002	
No	7.9 (1.7 – 21.4)	1			17.2 (8.9 – 28.7)	1		
L V Hypertrophy								
Yes	29.4 (17.5 – 43.8)	3.47 (0.91 – 13.3)	0.049		32.4 (21.5 – 44)	2.49 (0.84 – 7.5)	0.093	
No	10.7 (2.3 – 28.2)	1			16.1 (5.5 – 33.7)	1		
Ejection Fraction 								
< 55%	45.5 (28.1 – 63.6)	11.9 (3.1 – 46.4)	<0.001		50 (31.3 – 68.7)	4.75 (1.84 – 12.3)	0.001	
≥55%	6.5 (1.4 – 17.9)	1			17.4 (9.3 – 28.4)	1		
Left Heart Disease*								
Yes	54.2 (32.8 – 74.4)	11.8 (3.48 – 40.1)	<0.001		70.6 (44 – 89.7)
	10.7 (3.3 – 35)	<0.001	
No	9.1 (3 – 20)	1			18.3 (10.6 – 28.4)	1		
*Left Ventricular Hypertrophy with Low ejection fraction and Left Atrial Enlargement
BMI: Body Mass Index, BP: Blood Pressure, NC: Not Computed, OR: Odds Ratio, aOR: Adjusted OR, CI: Confidence Interval 	
