# Supplementary material for: Prevalence and determinants of pulmonary hypertension in a group of Cameroonian patients without chronic lung disease: a cross-sectional echocardiographic study
Source: BMC Res Notes. 2017 Nov 7;10:571. doi: 10.1186/s13104-017-2903-3 (PMC5678771; doi:10.1186/s13104-017-2903-3)
Supplement: Supplementary file 2 — Additional file 2: Table S2. Prevalence and risk factors (unadjusted and adjusted) of pulmonary hypertension in the general population. [file 13104_2017_2903_MOESM2_ESM.rtf]

Additional file 2: Table S2: Prevalence and risk factors (unadjusted and adjusted) of pulmonary hypertension in the general population
Variable	Prevalence  of Pulmonary Hypertension, (%, 95% CI)		Unadjusted Odds		Adjusted Odds	
			OR (95% CI)	p value		OR (95% CI)	p value	
Age								
≥ 55 years	30.1 (22.4 – 38.6)		3.44 (1.27 – 9.36)	0.005		3.17 (1.05 – 9.6)	0.014	
<55 years	11.1 (3.7 – 24.1)		1			1		
BMI 								
≥ 30 kg/m2	17 (8.1 – 29.8)		0.51 (0.22 – 1.14)	0.068		0.38 (0.13 – 1.09)	0.032	
<30 kg/m2	28.8 (21.1 – 37.6)		1			1		
Sex								
Male	22.8 (14.1 – 33.6)		0.79 (0.4 – 1.56)	0.251		NC	NC	
Female	27.3 (18.8 – 37.1)		1					
Systolic BP								
≥ 140 mmHg	29 (21.6 – 37.3)		2.83 (1.04 – 7.82)	0.024		4.23 (1.31 – 13.6)	0.032	
<140 mmHg	12.5 (4.2 – 26.8)		1			1		
Diastolic BP								
≥ 90 mmHg	26.7 (19.4 – 35.2)		1.35 (0.61 – 3)	0.237		NC	NC	
<90mmHg	21.3 (10.7 – 35.7)		1					
Pulse pressure								
≥ 65 mmHg	24.4 (15.3 – 35.4)		0.92 (0.46 – 1.82)	0.404		NC	NC	
<65 mmHg	26 (17.7 – 35.7)		1					
Mean BP 								
≥ 150 mmHg	22.4 (12.5 – 35.3)		0.79 (0.38 – 1.66)	0.276		NC	NC	
<150 mmHg	26.7 (19 -35.5)		1					
Left Atrial Enlargement								
Yes	40.8 (29.6 – 52.7)		4.33 (2.1 – 8.95)	<0.001		3.72 (1.7 – 8.17)	0.002	
No	13.7 (7.7 – 22)		1			1		
LV Hypertrophy								
Yes	31.1 (22.9 – 40.2)		3.49 (1.45 – 8.38)	0.002		2.68 ( 1.1 – 6.53)	0.011	
No	13.6 (6 – 25)		1			1		
Ejection Fraction 								
< 55%	46.8 (34 – 59.6)		6.06 (2.9 – 12.6)	<0.001		7.1 (3.2 – 15.8)	<0.001	
≥55%	13.8 (8.1 – 21.4)		1			1		
Left Heart Disease*								
Yes	61 (44.5 – 75.8)		9.14 (4.2 – 20.1)	<0.001		10.7 (4.6 – 24.6)	<0.001	
No	14.6 (9.2 – 21.6)		1			1		
*Left Ventricular Hypertrophy with Low ejection fraction and Left Atrial Enlargement
BMI: Body Mass Index, BP: Blood Pressure, NC: Not Computed, OR: Odds Ratio, aOR: Adjusted OR, CI: Confidence Interval	
