# Supplementary material for: Prevalence and determinants of pulmonary hypertension in a group of Cameroonian patients without chronic lung disease: a cross-sectional echocardiographic study
Source: BMC Res Notes. 2017 Nov 7;10:571. doi: 10.1186/s13104-017-2903-3 (PMC5678771; doi:10.1186/s13104-017-2903-3)
Supplement: Supplementary file 3 — Additional file 3: Table S3. Determinants of pulmonary hypertension without chronic lung disease. [file 13104_2017_2903_MOESM3_ESM.docx]

| Additional file 3: Table S3: Determinants of Pulmonary Hypertension without Chronic Lung Disease | | | | |
| --- | --- | --- | --- | --- |
| Determinant | Unadjusted Odds | | Adjusted Odds | |
|  | OR (95% CI) | *p* value | aOR (95% CI) | *p* value |
| Age ≥ 55 years | 3.44 (1.27 – 9.36) | 0.005 | 3.17 (1.05 – 9.6) | 0.014 |
| BMI ≥ 30 kg/m^2^ | 0.51 (0.22 – 1.14) | 0.068 | 0.38 (0.13 – 1.09) | 0.032 |
| Male sex | 0.79 (0.4 – 1.56) | 0.251 | NC | NC |
| Systolic BP ≥ 140 mmHg | 2.83 (1.04 – 7.82) | 0.024 | 4.23 (1.31 – 13.6) | 0.032 |
| Diastolic BP ≥ 90 mmHg | 1.35 (0.61 – 3) | 0.237 | NC | NC |
| Pulse pressure ≥ 65 mmHg | 0.92 (0.46 – 1.82) | 0.404 | NC | NC |
| Mean BP ≥ 150 mmHg | 0.79 (0.38 – 1.66) | 0.276 | NC | NC |
| Left Atrial Enlargement | 4.33 (2.1 – 8.95) | <0.001 | 3.72 (1.7 – 8.17) | 0.002 |
| Left Ventricular Hypertrophy | 3.49 (1.45 – 8.38) | 0.002 | 2.68 ( 1.1 – 6.53) | 0.011 |
| Ejection Fraction < 55% | 6.06 (2.9 – 12.6) | <0.001 | 7.1 (3.2 – 15.8) | <0.001 |
| Left Heart Disease* | 9.14 (4.2 – 20.1) | <0.001 | 10.7 (4.6 – 24.6) | <0.001 |
| *Left Ventricular Hypertrophy with Low ejection fraction and Left Atrial Enlargement  BMI: Body Mass Index, BP: Blood Pressure, NC: Not Computed, OR: Odds Ratio, aOR: Adjusted OR, CI: Confidence Interval | | | | |
